# Single Domain Antibodies as Carriers for Intracellular Drug Delivery: A Proof of Principle Study

**DOI:** 10.3390/biom11070927

**Published:** 2021-06-22

**Authors:** Sebas D. Pronk, Erik Schooten, Jurgen Heinen, Esra Helfrich, Sabrina Oliveira, Paul M. P. van Bergen en Henegouwen

**Affiliations:** 1 Research and Development Department, LinXis BV, 1081 GM Amsterdam, The Netherlands; pronk@linxispharmaceuticals.com (S.D.P.); schooten@linxispharmaceuticals.com (E.S.); 2 Cell Biology, Neurobiology and Biophysics, Department of Biology, Science Faculty, Utrecht University, 3584 CH Utrecht, The Netherlands; jurgen.heinen@student.uva.nl (J.H.); esra.helfrich@student.hu.nl (E.H.); p.vanbergen@uu.nl (P.M.P.v.B.e.H.); 3 Pharmaceutics, Department of Pharmaceutical Sciences, Science Faculty, Utrecht University, 3584 CG Utrecht, The Netherlands

**Keywords:** single-domain antibody, internalization, intracellular drug delivery, ADC

## Abstract

Antibody-drug conjugates (ADCs) are currently used for the targeted delivery of drugs to diseased cells, but intracellular drug delivery and therefore efficacy may be suboptimal because of the large size, slow internalization and ineffective intracellular trafficking of the antibody. Using a phage display method selecting internalizing phages only, we developed internalizing single domain antibodies (sdAbs) with high binding affinity to rat PDGFRβ, a receptor involved in different types of diseases. We demonstrate that these constructs have different characteristics with respect to internalization rates but all traffic to lysosomes. To compare their efficacy in targeted drug delivery, we conjugated the sdAbs to a cytotoxic drug. The conjugates showed improved cytotoxicity correlating to their internalization speed. The efficacy of the conjugates was inhibited in the presence of vacuolin-1, an inhibitor of lysosomal maturation, suggesting lysosomal trafficking is needed for efficient drug release. In conclusion, sdAb constructs with different internalization rates can be designed against the same target, and sdAbs with a high internalization rate induce more cell killing than sdAbs with a lower internalization rate in vitro. Even though the overall efficacy should also be tested in vivo, sdAbs are particularly interesting formats to be explored to obtain different internalization rates.

## 1. Introduction

Antibody-drug conjugates (ADCs) are considered to be among the most promising approaches to treat cell specific disorders. By making use of a targeting moiety which binds to a molecular target, a (cytotoxic) drug can be delivered specifically to diseased cells. ADCs consist of a small molecule drug which is attached via a linker molecule to the targeting moiety, for which antibodies are mostly used. Unless an extracellularly cleavable linker is employed, the ADC should internalize into the target cell, traffic to the lysosomes where the low pH and presence of lysosomal enzymes such as Cathepsin B release the drug from the targeting moiety [1,2,3,4]. Only then the drug can reach its action site, such as the microtubules. Therefore, internalization speed and intracellular trafficking of an ADC may have considerable impact on the functionality and effectivity of an ADC [5].

While conventional antibodies are often used as a targeting moiety, they come with several limitations: they are large in size and cannot easily extravasate and penetrate tissue to reach all the target cells [6,7]. Moreover, the internalization rate and intracellular fate of non-engineered antibodies may not be optimal to serve as targeting moiety for intracellular drug delivery [8]. 

Recent studies have suggested that small sized (15 kDa) single domain antibodies (sdAbs) are promising candidate targeting moieties for intracellular drug delivery. The sdAbs used here are VHHs, the variable domain of heavy chain only antibodies derived from the camelid family [9]. They can access and bind very small epitopes with high affinity and are able to reach small immunogenic pockets [10]. An epidermal growth factor receptor (EGFR) targeted by a half-life extended biparatopic sdAb conjugated to a platinum prodrug efficiently inhibited the growth of EGFR positive tumors [11]. In another recent study, a single dose administration of a half-life extended sdAb targeting the human epidermal growth factor receptor 2 (HER2) conjugated to Auristatin F demonstrated excellent efficacy in vivo and led to durable tumor remissions (>124 days) [12]. Another study compared a monoclonal antibody and sdAbs targeting tumors expressing prostate-specific membrane antigen (PSMA) conjugated to a potent DNA-alkylating agent. The authors showed that when the internalization speed was similar, the in vivo efficacy of the smaller sdAb was exceeding the monoclonal antibody [13]. These results are in line with the rationale that smaller sized targeting moieties such as sdAbs enable better drug delivery than full-sized antibodies.

Here, we selected sdAbs against the rat platelet derived growth factor β receptor (PDGFRβ) transmembrane receptor as a model target because it is reported to be overexpressed in different types of diseases, such as non-small-cell lung cancer, esophageal cancer, gastric cancer and human muscular dystrophy [14,15,16,17,18]. For this proof of principle study, we aimed to select internalizing sdAbs with either high or low internalization rates and to combine them into bivalent and biparatopic formats and investigate their internalization kinetics and drug delivery capacity. 

Interestingly, our results show that monovalent sdAbs, targeting the same receptor, can be selected with different internalization rates. After conjugation of the different sdAb formats to a cytotoxic drug, we demonstrated that the targeting moieties deliver the drugs to the lysosomal cell compartment and that targeting moieties with a higher internalization rate show improved in vitro efficacy compared to the targeting moieties with a lower internalization rate.

## 2. Materials and Methods

### 2.1. Cell Lines

The mouse squamous cell carcinoma cell line (SCCVII) was kindly provided by prof. dr. Lukas Mach, of the University of Natural Resources and Life Sciences in Vienna, Austria. These SCC cells were stably transfected with rat PDGFRβ-GFP (SCCVII-rPDGFRβ) and clone sorted for high GFP signal by FACS. The cell lines were cultured in Dulbecco’s Modified Eagle’s Medium (DMEM) (Lonza, Basel, Switzerland) with 100 units/mL streptomycin, 0.1 mg/mL penicillin and 10% fetal bovine serum (FBS). To maintain transgene expression, the cells were kept under selection pressure with 400 µg/mL G418 Sulfate (Thermo Fisher Scientific, Bleiswijk, The Netherlands). Cells were kept at 37 °C in a humidified atmosphere containing 5% CO_2_ and were repetitively tested negative for Mycoplasma.

### 2.2. Construction of Immune sdAb Phagemid Libraries

Immunization of two llamas was performed at Eurogentec (Seraing, Belgium). Animals received four injections of rat PDGFRβ protein ectodomain (25 μg per injection; Sino-Biological, Eschborn, Germany) with intervals of 2 weeks. Four weeks after the last interval, a final boost injection with SCCVII-rPDGFRβ (10^8^ cells) was administered. Llama serum was obtained, and the total mRNA isolated from peripheral blood lymphocytes was transcribed to cDNA. Specific primers were used to amplify the variable domain of the heavy chain only antibodies, which were eventually ligated into a phagemid vector as described previously [19]. Transformation of electrocompetent TG1 cells resulted in the generation of two libraries of approximately 10^6^–10^7^ clones, which were further used for phage display.

### 2.3. Evaluation of Anti-Rat PDGFRβ Immune Response in Llama Sera

Recombinant rat PDGFRβ (100 ng per well, Sino-Biological, Eschborn, Germany) was coated overnight at 4 °C in PBS in 96-well flat bottom plates (Nunc MaxiSorp™, Thermo Fisher Scientific, Bleiswijk, the Netherlands). Next day, plates were washed with PBS and blocked with blocking buffer (PBS, supplemented with 4% (*w*/*v*) skimmed milk powder (Merck Millipore, Darmstadt, Germany) for 1 h at room temperature (RT). Serial dilutions of pre-immune and immune sera were added in 2% MPBS (PBS, supplemented with 2% (*w*/*v*) skimmed milk powder (Merck Millipore, Darmstadt, Germany)). Detection of bound antibody was performed by incubation with rabbit anti-llama Ig antiserum (in 2% MPBS; Jackson Immunoresearch, West Grove, PA, USA) and horseradish peroxidase-conjugated donkey anti-rabbit Ig (in 2% MPBS; Jackson Immunoresearch, West Grove, PA, USA). All incubations were carried out for 1 h at RT and after every incubation, plates were washed four times with PBS. For quantification, 3,3′,5,5′-tetramethylbenzidine (TMB) substrate was added and the OD of the solution was measured at 450 nm.

### 2.4. Phage Display Selection of Internalizing Rat PDGFRβ Specific sdAbs

To isolate rat PDGFRβ sdAbs, two consecutive phage panning rounds were performed. Phages were first panned on purified protein, followed by a second panning round on SCCVII-rPDGFRβ cells. Phage selection in the first panning round was performed as described previously [19] with the only difference being the amount of coated protein which was 0.05 µg/mL of PDGFRβ protein. In the second panning round, 2.5 × 10^5^ SCCVII-rPDGFRβ cells were used. To skew selection towards sdAbs with internalizing ability, bio-panning was performed for 1 h at 37 °C, and the phages that bound to the membrane of SCCVII-rPDGFRβ cells were removed using an acid wash. Subsequently, the SCCVII-rPDGFRβ cells were lysed and the remaining internalized phages were used for *E. coli* TG1 cells infection. A colony PCR was performed to determine the number of clones containing a sdAb insert. Identifying unique clones in the selected library was accomplished with HinfI digestion (Thermo Fisher Scientific, Bleiswijk, The Netherlands).

### 2.5. sdAbs Production and Purification

#### 2.5.1. For Direct Use of the Periplasmic Fraction

In a deep well plate, 1.5 mL LB medium 0.2% (*w*/*v*) glucose with 100 μg/mL ampicillin was inoculated with *E. coli* TG1 containing the sdAb in the phagemid vector and incubated for 3 h at 37 °C at 180 rpm. The culture was then induced with 1 mM IPTG for overnighy sdAb production. Next morning, the culture was harvested by spinning down at 5000× *g* at 4 °C for 20 min. The pellet was resuspended in 100 µL PBS and freeze-thawed twice to release periplasmic content. The bacteria were then spun down at 5000× *g* at 4 °C for 20 min to collect the bacterial periplasm, which was then used directly in a binding assay on immobilized ectodomain. 

#### 2.5.2. For Purification

Initial productions and binding assays with the monomeric sdAbs were performed with the sdAbs in the original phagemid vector. The selected sdAb sequences were re-cloned in a modified pET21 to introduce a N-terminal pelB signal sequence and a C-terminal cysteine and EPEA tag. For productions, pre-cultures were made by inoculating *E. coli* BL21-DE3 Codonplus (Stragene, Bellingham, WA, USA) containing the sdAb in pET21 vector into 90 mL LB medium, 2% (*w*/*v*) glucose and 100 μg/mL ampicillin, which was incubated overnight at 37 °C at 180 rpm. The 5 L Eppendorf BioFlo^®^/CelliGen^®^ 115 fermentor containing probes for dissolved oxygen (DO), pH and temperature measurements was prepared according to the manufacturer’s instructions and all measurements were monitored via the BioCommand program. A 1:100 dilution of the overnight pre-culture was added to 5 L Terrific Broth (TB) medium, supplemented with 17 mM KH_2_PO_4_, 72 mM K_2_HPO_4_, 0.1% (*w*/*v*) glucose, 100 μg/mL ampicillin and 1:10,000 anti-foam. Bacteria were grown at 37 °C and OD_600_ was monitored using a spectrophotometer until the desired OD_600_ of ±1 was reached. Temperature was then lowered to 25 °C and the bacteria were induced with 1 mM IPTG for overnight sdAb production. Next morning, the culture was harvested by spinning down the culture at 5000× *g* at 4 °C for 20 min. The pellet was resuspended in 750 mL PBS and freeze-thawed twice to release periplasmic content. The bacteria were then spun down at 5000× *g* at 4 °C for 30 min to collect the bacterial periplasm, which was then filtrated through a 0.45 µm filter.

Periplasmic fraction containing EPEA-tagged sdAb was purified on the ÄKTAXpress chromatography system using a 1 mL CaptureSelect™ C-tag column (Thermo Fisher Scientific, Bleiswijk, The Netherlands) and 2 × 5 mL HiTrap Desalting Columns (GE Healthcare, Hoevelaken, The Netherlands). Sample was loaded (1 mL/min) onto the C-tag column, after which bound sdAb was eluted using 2 mM Tris, 2 M MgCl_2_, pH 7 and buffer exchanged to PBS using the HiTrap Desalting columns. Purified fractions were loaded on with SDS-PAGE gels for purity assessment and stored at −80 °C.

### 2.6. Conjugations

#### 2.6.1. sdAb—Maleimide—Fluorophore Conjugation

The free C-terminal cysteine in the sdAbs was used for site-directed maleimide-fluorophore conjugation using a procedure described elsewhere [20]. Briefly, sdAbs were incubated with 2 molar equivalents of TCEP in borate buffer (25 mM sodium borate pH 8, 25 mM NaCl, 1 mM DTPA) at 37 °C for 2 h. The maleimide-IRDye800CW (LI-COR Biosciences, Lincoln, NE, USA) or maleimide-AlexaFluor647 (Thermo Fisher Scientific, Bleiswijk, The Netherlands) was added at 5–10 molar equivalents and incubated on ice for 1 h. The conjugates were purified from free fluorophore using two consecutive 2 mL Zeba spin desalting columns (Thermo Fisher Scientific, Bleiswijk, The Netherlands) which were pre-equilibrated with PBS. The amount of free dye in the samples was determined by SDS-PAGE. Upon gel electrophoresis, fluorescence was detected with an Odyssey infrared scanner at 700 nm or 800 nm. The degree of conjugation (DoC) was determined following the manufacturer’s protocol by measuring the absorbance at 280 and 650 nm for AlexaFluor647 and 280 and 800 nm for IRDye800 using a Nanodrop spectrophotometer (Nanodrop Technologies, Wilmington, DE, USA).

#### 2.6.2. sdAb—Lx—Auristatin F Conjugation

The free C-terminal cysteine in the sdAbs was used for site-directed Auristatin F conjugation using the Lx linker technology as described elsewhere [21]. Briefly, sdAbs were incubated with 2 molar equivalents of TCEP in borate buffer (25 mM sodium borate pH 8, 25 mM NaCl, 1 mM DTPA) at 37 °C for 2 h. The AF-Lx-thiourea (prepared by mixing AF-Lx-I and 20 mM thiourea (1:1) at 37 °C for 2 h) was added to the reduced sdAbs and incubated at 37 °C for 1 h. The conjugates were purified using 10 kDa Amicon Ultra centrifugal filters (Merck Millipore, Darmstadt, Germany). The purity and degree of conjugation (DoC) of the conjugates was determined by SEC-MS.

### 2.7. Binding Assays

#### 2.7.1. On Immobilized Ectodomain

Purified rat PDGFRβ was purchased from Sino-Biological (Eschborn, Germany) and coated overnight at 4 °C at 1 μg/mL in PBS in ELISA plates (Nunc MaxiSorp™, Thermo Fisher Scientific, Bleiswijk, the Netherlands). Next day, plates were washed with PBS and blocked with blocking buffer (PBS, supplemented with 4% (*w*/*v*) skimmed milk powder (Merck Millipore, Darmstadt, Germany)) for 1 h at RT. Next, three-fold serial sdAb dilutions in PBS starting from 500 nM were added. All incubations were carried out for 1 h at RT and after every incubation, plates were washed four times with PBS. The fluorescently labelled sdAbs were detected directly, or via indirect detection using an incubation with rabbit anti-VHH (clone QE19, 1:2000; QVQ BV, Utrecht, The Netherlands), followed by an IRDye800conjugated goat-anti-rabbit antibody (1:2000, LI-COR Biosciences, Lincoln, NE, USA). The plate was scanned using the Odyssey near-infrared scanner (LI-COR Biosciences, Lincoln, NE, USA).

#### 2.7.2. On Cells

SCCVII or SCCVII-rPDGFRβ cells (approximately 10^4^ cells per well) were seeded in a Nunclon™ Delta Surface 96-wells tissue culture plate (Thermo Fisher Scientific, Bleiswijk, The Netherlands) and allowed to adhere overnight. Three-fold serial sdAb dilutions were added onto cells in binding medium (DMEM without phenol red, supplemented with 25 mM HEPES, 1% *w*/*v* bovine serum albumin, pH 7.2) at 4 °C to prevent internalization. After 2 h of incubation, the sdAb was removed and cells were fixed by incubating the cells in 4% PFA for 20 min at RT. Detection of sdAbs was performed as described above.

### 2.8. Competition Assay on Immobilized Ectodomain

Purified rat PDGFRβ (Sino-Biological, Eschborn, Germany) was coated overnight at 4 °C at 1 μg/mL in PBS in ELISA plates (Nunc MaxiSorp™, Thermo Fisher Scientific, Bleiswijk, The Netherlands). Next day, plates were washed with PBS and blocked with PBS, supplemented with 4% (*w*/*v*) skimmed milk powder (Merck Millipore, Darmstadt, Germany) and 0.05% *v/v* Tween, for 1 h at RT. All further incubations were carried out for 1 h at RT in PBS supplemented with *v/v* 0.05% Tween (PBST). A fixed concentration of IRDye800 conjugated sdAb was mixed with unconjugated competitors (sdAbs or ligand) in three-fold serial dilutions, starting with a 20-fold molar excess. Fluorescent signal of the IRDye800 conjugated sdAb was detected using the Odyssey near-infrared scanner (LI-COR Biosciences, Lincoln, NE, USA).

### 2.9. Internalization Assay

SCCVII-rPDGFRβ cells (approximately 10^4^ cells per well) were seeded in a 96-wells tissue culture plate (Nunclon™ Delta Surface, Thermo Fisher Scientific, Bleiswijk, The Netherlands) and allowed to adhere overnight. Next day, internalization of the IRDye800 conjugated sdAb (5 nM) into the cells was measured after an incubation at 37 °C for period of 15 min. Total fluorescence as well as fluorescence attributed to the internalized fraction were measured and used to calculate the internalization rate constant as described by Heukers et al. [22]. The internalized fraction is assessed by performing two subsequent acid washes (0.2 M glycine-HCl, 150 mM NaCl, pH 2.3) removing the membrane bound sdAb fraction from the cells. For the cell loading assays, the same procedure was applied with minor modifications. Longer timepoints (up to 72 h) were measured and only the internalized fraction was plotted.

### 2.10. PDGFRβ Activation Assay

SCCVII-rPDGFRβ cells (approximately 10^5^ cells per well) were seeded in a 12-wells tissue culture plate (Nunclon™ Delta Surface, Thermo Fisher Scientific, Bleiswijk, The Netherlands) and allowed to adhere overnight. The next day, the medium was refreshed with medium containing 10 ng/mL of the PDGFRβ ligand (PDGF-BB) and/or 10 nM PDGFRβ specific sdAbs. After 15 min of incubation at 37 °C, cells were cooled down on ice and washed twice with ice-cold PBS. Cell lysates were prepared by taking the cells up in 30 μL 1× Laemmli protein sample buffer without DTT. Next, 20 µL lysates were boiled for 10 min at 100 °C, loaded on a 8–12% (*w*/*v*) PAGE gel (Bio-rad, Veenendaal, the Netherlands) and blotted onto a PVDF membrane (Roche, Mannheim, Germany). Blots were stained overnight for phosphorylated PDGFRβ using a rabbit polyclonal antibody targeting the Tyr751 phosphorylated receptor (Cell Signaling Technology, Leiden, The Netherlands), followed by a 1 h incubation with IRDye800-conjugated goat-anti-rabbit secondary antibody (LI-COR Biosciences, Lincoln, NE, USA). As a loading control, the blots were also stained for actin using a monoclonal mouse-anti-actin antibody (Sigma-Aldrich, Zwijndrecht, The Netherlands), followed by a 1 h incubation with an IRDye680-conjugated goat-anti-mouse secondary antibody (LI-COR Biosciences, Lincoln, NE, USA). Fluorescent signal was detected using the Odyssey near-infrared scanner (LI-COR Biosciences, Lincoln, NE, USA).

### 2.11. Confocal Fluorescence Microscopy 

SCCVII-rPDGFRβ cells were seeded on cover slips in a 24-wells tissue culture plate (approximately 10^4^ cells per well) and allowed to adhere overnight. The next day, cells were incubated for 2 h at 37 °C with 5 nM AlexaFluor647-conjugated sdAbs in binding medium (DMEM without phenol red, supplemented with 25 mM HEPES, 1% *w*/*v* bovine serum albumin, pH 7.2). 60 nM lysoTracker™ Red DND-99 (ex 577 nm, em 590 nm) (Thermo Fisher Scientific, Bleiswijk, the Netherlands) or 1 mg/mL TMR-Amino Dextran 70 kDa (ex 555 nm, em 580 nm) (Fina Biosolutions LLC, Rockville, MD, USA) were co-incubated with the AlexaFluor647-conjugated sdAb conjugates (ex 650 nm, em 668 nm). Upon incubation, cells were washed with binding medium and PBS. Afterwards, the cells were fixed in 4% PFA and PFA-induced autofluorescence was quenched with 50 mM glycine in PBS for 15 min at RT. Nuclei were stained with DAPI (ex 358 nm, em 461 nm) (Thermo Fisher Scientific, Bleiswijk, the Netherlands) for 5 min and after washing, the cover slips were mounted using Mowiol (Merck Millipore, Darmstadt, Germany). Images were taken using a Zeiss LSM700 confocal microscope (Carl Zeiss Microscopy GmbH, Germany) equipped with a 63× oil immersion objective. Three images were taken, after which a representative was included in the figure. All images are taken with the same microscope settings and no image editing has been performed.

### 2.12. Mass Spectometry Analysis of sdAb-Auristatin F Conjugates

LC-MS analysis was performed using a Thermo Finnigan LC system (Thermo Finnigan, San Jose, CA, USA) coupled to a Bruker Q-TOF mass spectrometer (Bremen, Germany) equipped with an electrospray ionization (ESI) source. Mass determination was performed using a Zenix-C column (4.6 × 300 mm; 5 μm; Sepax Technologies Inc., Newark, DE, USA). The mobile phase consisted of a mixture of water, acetonitrile, trifluoroacetic acid and formic acid (79.9/19.9/0.1/0.1, *v*/*v*/*v*/*v*, respectively). A 17-min isocratic run was performed, and MS analysis was achieved in positive ionization mode. The protein ion charge assignment and molecular mass determinations were performed using the “Charge Deconvolution” utility of Bruker Daltonics Data Analysis software.

### 2.13. Cell Viability Assays

Cell viability assays were performed using the RealTime-Glo™ MT Cell Viability assay (Promega, Leiden, The Netherlands) according to manufacturer’s protocol or using the CellTiter-Blue^®^ assay (Promega, Leiden, The Netherlands) according to manufacturer’s protocol. As starting points, 750 cells/well were seeded in an opaque walled white 96-well plate to maintain assay linearity. SCCVII or SCCVII-rPDGFRβ were incubated with different sdAb-Lx-Auristatin F constructs and monitored for 72 h by measuring the luminescence or fluorescence using the FLUOstar OPTIMA FL microplate reader (BMG LABTECH). When the cell viability assay was performed in the presence of lysosomal exocytosis inhibitor vacuolin-1, the same procedure was applied with minor modifications. In a separate experiment cells were pre-incubated for 3 h with 1 µM vacuolin-1 (Sigma-Aldrich, Zwijndrecht, The Netherlands), after which the medium was refreshed with medium containing the sdAb-drug conjugates and 1 µM vacuolin-1.

### 2.14. Receptor Quantification on SCCVII-rPDGFRβ Cells

A binding assay on cells and a titration series of IRDye800 conjugated PDGFRβ binding sdAb (3G7) was performed in parallel. The binding assay on cells was performed as described before, with the modification that the cells were lysed in 100 µL RIPA buffer before the plate was scanned.

In the titration series, a concentration range of the IRDye800 conjugated sdAb was made starting at 10 nM and ending at 1.2 × 10^−3^ pM in a volume 100 µL. The B_max_ of the binding assay was interpolated with the obtained titration series to calculate the number of fluorescently bound molecules per well. To determine the average number of cells per well, cells were harvested and counted. The number of fluorescent molecules per well was then divided by the number of cells per well to obtain the number of fluorescent molecules per cell and thus the number of receptors localized on the cell membrane per cell.

### 2.15. Live Cell Imaging Using Spinning Disc Microscopy

SCCVII-rPDGFRβ cells were seeded in a glass bottom µ-slide 8-well chambered coverslip (Ibidi, Gräfelfing, Germany) (approximately 10^4^ cells per well) and allowed to adhere overnight at 37 °C, 5% CO_2_ in a humidified incubator. The next day, the cells were transferred the TokaiHit incubation chamber of the microscope to keep the cells at 37 °C. At the starting point of the timelapse-experiment, 10 nM sdAb conjugated to maleimide pHrodo™ Red (Thermo Fisher Scientific, Bleiswijk, The Netherlands) was added to the cells. Live-cell imaging was performed using a using a Nikon Eclipse Ti confocal spinning disc microscope equipped with Perfect Focus System and a 60× oil objective. Metamorf software was used to make a time-lapse video (picture every 15 sec with 250 ms exposure time). Acquired files were background corrected and time stamped with Fiji/ImageJ software.

## 3. Results

### 3.1. Induction of a Humoral Anti-PDGFRβ Response in Llama Glama

To obtain PDGFRβ specific sdAbs, two llamas were immunized with rat PDGFRβ ectodomain (ECD) and SCCVII cells transfected with rat PDGFRβ (SCCVII-rPDGFRβ) with approximately 4 × 10^5^ receptors on the cell membrane (Figure S1).

**Figure 1:**
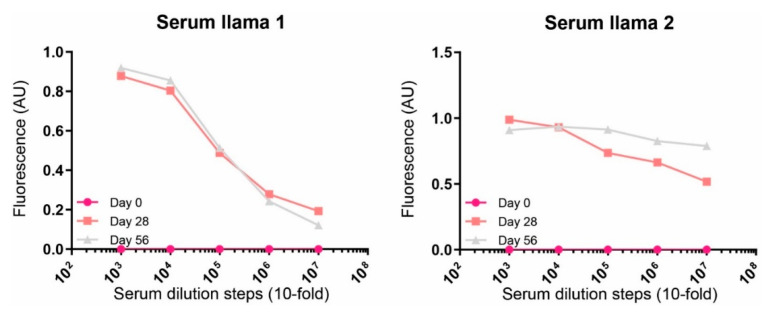
Detection of rat PDGFRβ binding antibodies in llama serum. Plates were coated with rat PDGFRβ ECD. Serial dilutions of the sera obtained at days 0 (before immunization) and 28 and 56 (during immunization) were plated onto the PDGFRβ coated wells.

To test whether the rat PDGFRβ immunized llamas developed a specific immune response, serum was obtained from the animals at days 0, 28 and 56. Levels of rat PDGFRβ specific antibodies were detected using an ELISA setup. A clear increase in detected antibodies bound to PDGFRβ was observed in the post-immunization samples indicating that an immune response against rat PDGFRβ had successfully been elicited in both llamas (Figure 1). The serum from day 56 was used for the construction of two sdAb phage libraries with sizes around 10^6^–10^7^ colony forming units (Table S1).

### 3.2. Selection, Characterization and Design of sdAb Constructs

To select for internalizing rat PDGFRβ specific sdAbs, two consecutive phage panning rounds were performed. Phages were first panned on purified rat PDGFRβ protein, followed by a second panning round on SCCVII-rPDGFRβ cells. To skew selection towards sdAbs with internalizing ability, the second bio-panning was performed at 37 °C allowing phages to internalize into the target cells. Only the internalized PDGFRβ sdAb displaying phages were used in subsequent steps leading to 11 unique clones encoding internalizing sdAbs binding rat PDGFRβ.

**Figure 2:**
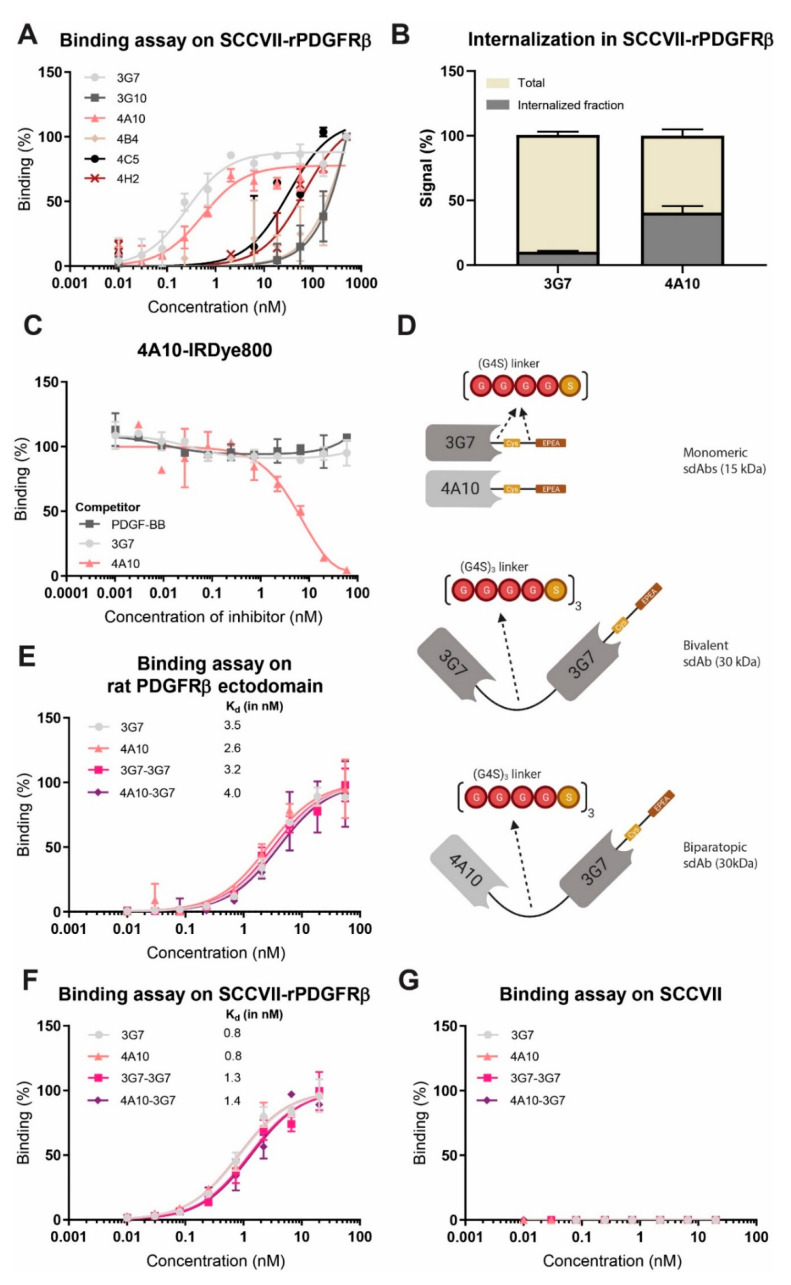
Characterization of rat PDGFRβ specific sdAb constructs, representative of at least 2 independent experiments. (**A**) Binding to SCCVII-rPDGFRβ cells with serial dilutions of the monomeric constructs. (**B**) Internalization assay with SCCVII-rPDGFRβ cells comparing the total and internalized sdAb fractions after a 30 min 37 °C incubation. (**C**) Competition assay in which 4A10 conjugated to IRDye800 (4A10-IRDye800) was incubated on immobilized rat PDGFRβ-ECD in the presence or absence of excess unconjugated competitor (sdAb or PDGF-BB). (**D**) Schematic illustration of (from top to bottom) monomeric sdAbs 3G7 and 4A10 (both 15 kDa), bivalent sdAb 3G7-3G7 (30 kDa) and biparatopic construct 4A10-3G7 (30 kDa). The sdAbs are fused by a flexible Gly-Ser (G4S)_3_ linker. The cysteine (the specific conjugation site) and EPEA affinity purification tag are located at the C-terminus. (**E**) Binding on immobilized rat PDGFRβ-ECD with serial dilutions of the designed constructs conjugated to IRDye800. (**F**) Binding to SCCVII-rPDGFRβ cells with serial dilutions of the designed constructs conjugated to IRDye800. (**G**) Binding to SCCVII cells with serial dilutions of the designed constructs conjugated to IRDye800.

**Figure 3:**
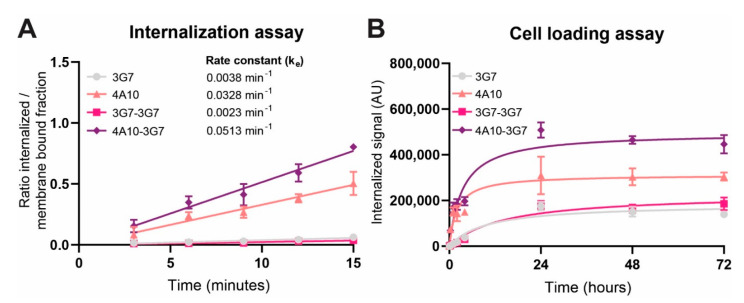
Quantitative characterization of sdAb-IRDye800 internalization, representative of 5 independent experiments. (**A**) Internalization assay comparing the internalization rates of sdAb constructs during a 15 min incubation at 37 °C. (**B**) Cell loading assay comparing the accumulation of fluorescent sdAb constructs during a 72 h incubation at 37 °C.

Based on the binding assays performed with bacterial periplasm containing the unpurified sdAbs, six sdAbs showed strong binding (Figure S2). These sdAbs, still in the phagemid vector, were produced, purified and further characterized, from which 3G7 and 4A10 were selected based on their high binding affinities (K_D_ < 10 nM) on immobilized ECD (Figure S3) and SCCVII-rPDGFRβ cells (Figure 2A). To determine the degree of internalization of these sdAbs, cells were incubated for 30 min at 37 °C. Interestingly, the two sdAbs displayed different results: 4A10 showed approximately 3-fold higher internalized fraction than 3G7 (Figure 2B). To investigate whether we could increase the internalization capacity of the slower internalizing sdAb, we constructed a bivalent construct with this sdAb, i.e., 3G7-3G7. As biparatopic sdAbs have been shown to be the most effective format for receptor mediated internalization [22], we evaluated whether 3G7 and 4A10 would be suitable candidates. To this end, the selected sdAbs were tested for binding to overlapping epitopes in a competition assay. A low concentration of 4A10, or 3G7 conjugated to IRDye800 (4A10-IRDye800 or 3G7-IRDye800) was incubated on immobilized rat PDGFRβ ECD in the presence or absence of an excess amount of each of the unconjugated competitor sdAb or the natural ligand of PDGFRβ (PDGF-BB). As expected, 4A10 competes with itself, validating this assay. The signal of 4A10-IRDye800 (Figure 2C) and 3G7-IRDye800 (Figure S4) did not decrease upon adding a surplus of PDGF-BB. Indicating both sdAbs do not bind to on the PDGF-BB binding site of PDGFRβ. Clearly, the signal of 4A10-IRDye800 did not decrease upon adding a surplus of unconjugated 3G7 (Figure 2C, vice versa Figure S4), suggesting that 3G7 binds another epitope than 4A10. These results indicate that 4A10 can be combined with 3G7 to develop a biparatopic sdAb construct, to investigate the effect on the internalization rate when the slow internalizing sdAb is fused to the fast internalizing sdAb.

Subsequently, 3G7 and 4A10, 3G7-3G7 and 4A10-3G7 were codon-optimized and cloned into a bacterial expression vector containing a C-terminal free cysteine for site directed conjugation and an EPEA tag for affinity purification. For the bivalent (3G7-3G7) and biparatopic (4A10-3G7) constructs, the sdAbs were separated by a flexible (G4S)_3_ linker (Figure 2D). The constructs were conjugated via the free cysteine to IRDye800 (the amount of free fluorophore was below 10% (Figure S5) in all samples, and the DoC was between 0.8–1.0). The binding affinity of the four IRDye800 conjugated sdAb constructs was determined on immobilized ECD and on SCCVII-rPDGFRβ cells, verifying that all sdAb constructs bind the target receptor with a K_D_ in the nanomolar range (Figure 2E,F), similar to the unconjugated sdAbs (Figure S6). The sdAb constructs were not binding to non-transfected SCCVII cells, confirming binding specificity (Figure 2G). Altogether, four sdAb constructs with a similar K_D_ were prepared for further testing.

### 3.3. The Designed sdAb Constructs Have Different Internalization Rates Which Is Predictive for sdAb Accumulation in Cells after Long Periods of Time

To determine the internalization speed of the different sdAb constructs, quantitative internalization assays with the same IRDye800 fluorescently labelled sdAb constructs were performed. 

4A10-3G7 clearly internalized the fastest (0.0513 min^−1^), followed by 4A10 (0.0328 min^−1^), while 3G7 and 3G7-3G7 showed relatively low internalization rates (0.0038 min^−1^ and 0.0023 min^−1^ respectively) (Figure 3A). Apparent is the difference in internalization rate between both monomers, which is in line with Figure 2B. 4A10 has a very high internalization rate, especially for a monomeric sdAb, while 3G7 internalizes with a relatively low internalization rate. Bivalent sdAb construct 3G7-3G7 does not enhance the internalization speed of monomer 3G7. However, addition of 3G7 to 4A10 in the biparatopic construct 4A10-3G7 does result in even faster internalization compared to 4A10 alone. 

To test whether this difference in internalization rate also has an effect on the total accumulation of sdAb in the cell after a longer period of time, a cell loading assay was performed. In this cell loading assay, target cells were incubated with the fluorescent sdAb constructs for 72 h. The total internalized sdAb fraction was measured to give an indication about how much sdAb accumulates in the cells over time. The results indicated that the internalization rate reflects the intracellular accumulation over a longer period of time. The biparatopic sdAb construct 4A10-3G7 accumulates to the highest degree in the cells after a 72 h, followed by 4A10 (Figure 3B). 3G7 and 3G7-3G7 show the lowest intracellular accumulation. After 24 h of incubation with the sdAbs, the fluorescent accumulation did not increase anymore, suggesting that the internalization kinetics of the fluorescent sdAbs reached an equilibrium after this period of time.

### 3.4. sdAb Constructs Do Not Interfere with the PDGFRβ Activation Status

Since the primary purpose of the sdAb constructs is to serve as targeting moieties to deliver drugs to PDGFRβ-positive cells, without inducing unwanted cell signaling, we investigated whether the sdAbs constructs could activate PDGFRβ, which is a tyrosine kinase. Briefly, SCCVII-rPDGFRβ were cultured in the presence or absence of the sdAb constructs. In parallel, cells were incubated with the natural ligand PDGF-BB as positive control for receptor activation. Upon incubation, the cells were lysed, and lysates were subjected to western blotting for detection of phosphorylated (phospho-)PDGFRβ. 

**Figure 4:**
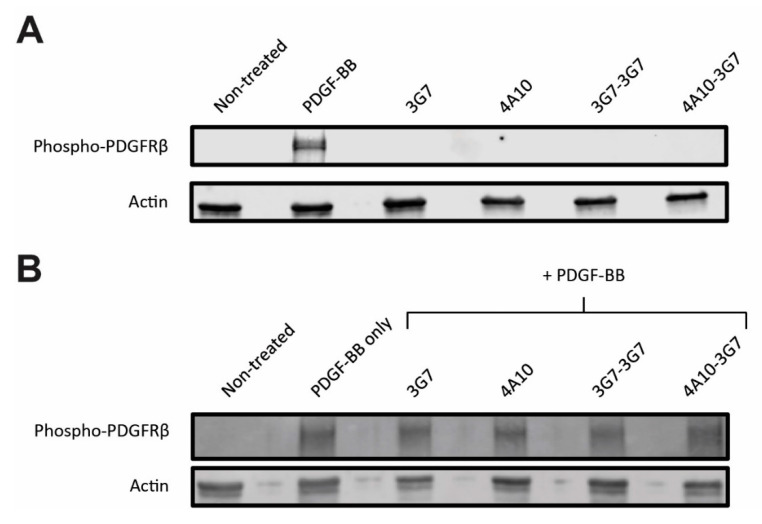
Activation assay investigating phosphorylation of PDGFRβ in SCCVII-rPDGFRβ cells, representative of 2 independent experiments. (**A**) SCC-rPDGFRβ cells were incubated with the natural ligand (PDGF-BB) or the sdAb constructs, after which phosphorylation of PDGFRβ is detected by western blot using a phospho-PDGFRβ specific antibody. Complete blot Figure S7A, quantification in Figure S7C. (**B**) SCC-rPDGFRβ cells were co-incubated with the sdAb constructs in presence of the natural ligand (PDGF-BB), after which phosphorylation of PDGFRβ is detected by western blot. Complete blot Figure S7B, quantification in Figure S7D.

As shown in Figure 4A, a clear increase of phospho-PDGFRβ was observed upon exposure to PDGF-BB, demonstrating that the receptor can be phosphorylated upon stimulation. No change in phospho-PDGFRβ levels was observed in cells exposed to the sdAbs (complete blot Figure S7A). Furthermore, none of the sdAbs inhibited phosphorylation of PDGFRβ by PDGF-BB, as displayed in Figure 4B (complete blot Figure S7B). This suggests that when the sdAbs bind PDGFRβ expressing cells, the normal PDGFRβ signaling cascade will not be affected.

### 3.5. All sdAb Constructs Internalize Mainly via the Macro-Pinocytotic Pathway and Traffic to Lysosomal Cell Compartments 

**Figure 5:**
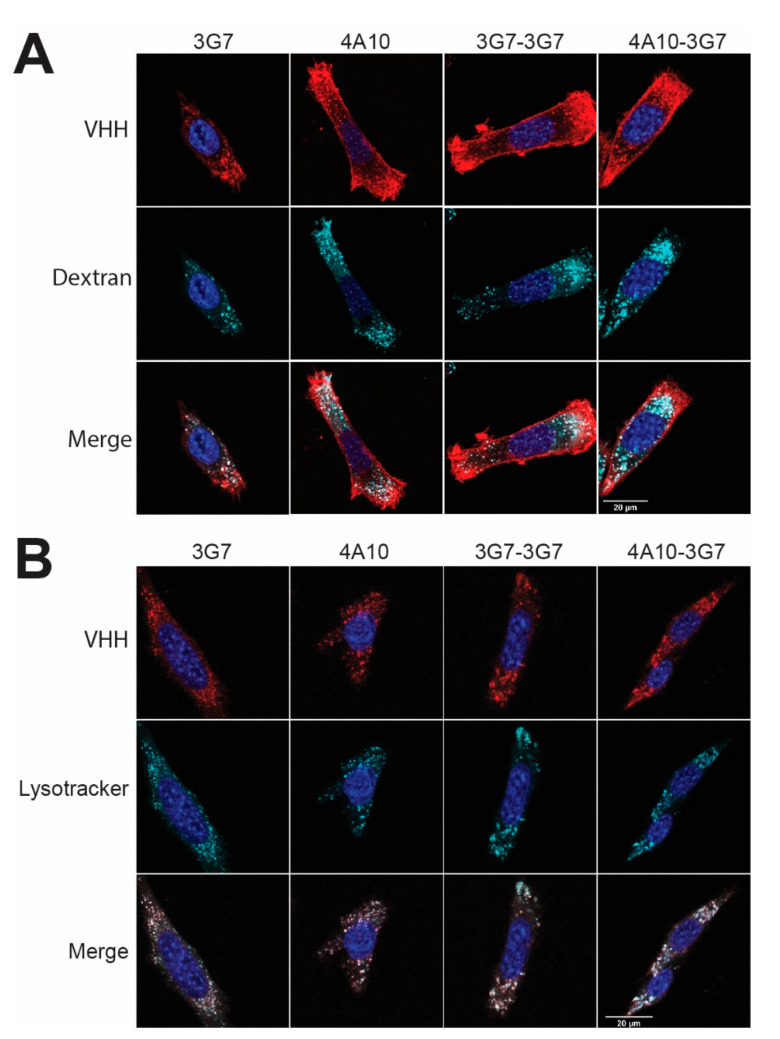
Imaging of AlexaFluor647 conjugated sdAb internalization in SCCVII-rPDGFRβ cells. Images were taken using a confocal microscope using a 63× objective. (**A**) AlexaFluor647 labelled sdAb constructs (in red) are co-incubated with fluorescent dextran (in cyan) to assess their mechanism of internalization. Scalebar is 20 µm. (**B**) AlexaFluor647 labelled sdAb constructs (in red) are co-incubated with Lysotracker™ Red (in cyan) to assess their colocalization (in white). Scalebar is 20 µm.

To investigate the intracellular trafficking of the sdAb constructs, confocal imaging experiments were performed. The target cells are incubated for 2 h at 37 °C with AlexaFluor647 conjugated sdAbs, which show similar DoC, free dye and binding affinity as the IRDye800 conjugated sdAbs (Figure S6). The sdAbs are co-incubated with 70 kDa dextran, a molecule known to internalize via macropinocytosis. Clear internalization of the fluorophore labelled sdAb constructs and the dextran was observed, as indicated by the punctuated pattern (Figure 5A, top rows). In fact, colocalization of the internalized sdAb constructs and dextran was observed (Figure 5A, bottom row), suggesting that all sdAb constructs internalize via the macro-pinocytotic pathway. 

To further specify the intracellular fate of the sdAb constructs, a co-staining with Lysotracker™ Red, a marker for lysosomes, was performed. Here, the target cells are co-incubated for 2 h at 37 °C with Lysotracker™ Red and fluorescently labelled sdAb. Colocalization has been observed for the Lysotracker and the fluorescently labelled sdAbs (Figure 5B), further supporting that the sdAbs are trafficking to lysosomal compartments upon internalization. To visually show internalization of the fastest internalizing sdAb constructs, a live cell imaging experiment was conducted using 4A10-3G7 conjugated to the pHrodo™ Red dye (Video S1). This dye only emits a fluorescent signal once it is in an acidic environment, such as the late endosomes and lysosomes. Clear internalization of 4A10-3G7 is observed.

### 3.6. sdAb Constructs with Different Internalization Rates Induce Different Levels of Cytotoxicity

**Figure 6:**
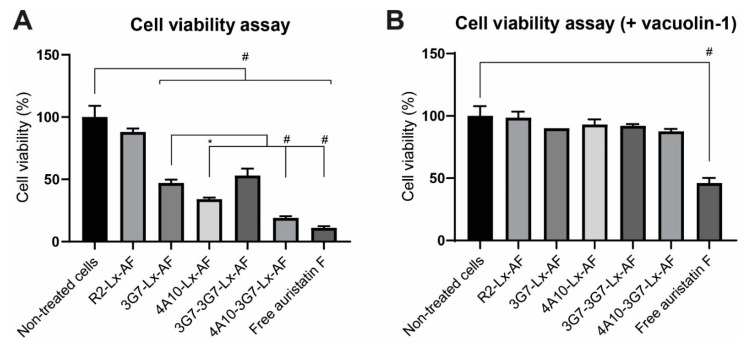
Cell viability assay on SCCVII-rPDGFRβ cells, representative of 3 independent experiments. (**A**) Cell viability after 72 h of treatment with 250 nM conjugates or free Auristatin F. *p* values were determined using a one-way ANOVA test. * *p* ≤ 0.05 and # *p* ≤ 0.001. (**B**) Same assay as in A, but in the presence of lysosomal exocytosis inhibitor vacuolin-1.

To investigate whether the difference in intracellular accumulation and internalization rate of the sdAbs also results in different efficacies when conjugated to a cytotoxic drug, the sdAbs were conjugated to Auristatin F (AF) (DoC ~0.7–0.8 in all samples, SEC-MS in Figure S8). AF is an anti-mitotic agent that inhibits cell division by blocking the polymerization of tubulin and has potent and selective antitumor activity when conjugated to a sdAb [12]. AF was conjugated to the sdAb constructs via a platinum-based Lx linker. In a recent study where this linker is used to allow stable coupling of AF to a HER2 specific sdAb, excellent and durable anti-tumor efficacy is shown. Furthermore, this linker should prevent the drug from being released outside the cell [12,21]. As a control, a non-binding sdAb named R2 [12,23], was conjugated to AF via the same linker and used to verify that the cytotoxicity is sdAb specific. The target cells were incubated over time with sdAb-drug conjugates and their cytotoxic effect was evaluated (Figure 6A).

The R2-Lx-AF conjugate did not show significant cell killing, indicating that a targeting moiety is needed for cytotoxicity. None of the AF-conjugates showed toxicity if tested on non-transfected SCCVII cells, showing specificity and stability of the conjugates (Figure S9). The conjugates have thus to be internalized via the target receptor before the payload can exert its function. Interestingly, 4A10-3G7-Lx-AF was the most cytotoxic conjugate, followed by the 4A10-Lx-AF. 3G7-Lx-AF and 3G7-3G7-Lx-AF also inhibited cell proliferation but were less potent than the other drug-conjugates. The results were considerably in line with the results from the internalization and loading assay: the sdAbs with the highest internalization rates were more cytotoxic as AF-conjugate, compared to sdAbs with lower internalization rates. 

The assay was repeated in the presence of vacuolin-1. Vacuolin-1 is a cell permeable molecule that alkalizes the lysosomal pH and therefore disrupts autophagosome-lysosome fusion and the endosomal-lysosomal degradation pathway [24]. As expected, free Auristatin F, which does not require receptor mediated internalization, still had a significant cytotoxic effect (Figure 6B). Clearly, the cytotoxicity of all conjugates was inhibited by co-incubation with vacuolin-1. These data further support the finding that all sdAb constructs traffic to the lysosomes upon internalization. 

## 4. Discussion

Recent studies are encouraging the development of smaller antibody-drug conjugates or sdAb-drug conjugates for their advantage in tissue distribution, and especially homogeneous targeting [12,13]. In this proof-of-principle study we investigate a panel of sdAbs targeting the same receptor but with different internalization rates to determine whether this correlates with the overall efficacy of intracellular drug delivery in vitro. 

Previous studies have shown that the internalization rate of antibodies can be affinity dependent [25,26]. In vitro internalization assays revealed that antibody internalization generally increased with affinity, plateauing once the rate of antigen internalization exceeded the rate of antibody dissociation [25]. In our study, as all investigated constructs have similar binding affinities, in the low nanomolar range, it is not expected that this will affect internalization (Figure 2E,F).

We tested internalizing monomeric, bivalent and biparatopic sdAb constructs and showed that biparatopic construct 4A10-3G7 clearly internalizes fastest, followed by the monomer 4A10. The internalization rate of these fast internalizing constructs even seem to be slightly higher than the reported internalization rate of the natural PDGFRβ ligand PDGF-BB, known to dimerize the receptor [27]. Monomer 3G7 and bivalent construct 3G7-3G7 internalize relatively slow compared to the other sdAb constructs. The difference in internalization speed between both monomers could indicate that internalization speed is a characteristic of the targeting moiety, possibly depending on the binding epitope, and not solely on the target receptor. 

An interesting observation is that addition of a slow internalizing sdAb to a fast-internalizing sdAb led to an even faster internalizing sdAb construct, biparatopic 4A10-3G7. In a recent study, a fast internalizing single-chain variable fragment (scFv) fused to a non-binding antibody was rendered slow internalizing by substitution of the non-binding antibody for a slow-internalizing antibody [28]. Based on this example, one could expect that the biparatopic sdAb construct would internalize slower than 4A10 alone. However, here the two sdAbs combined lead to increased internalization rates. 

Other studies have indicated that bivalent sdAbs may induce receptor dimerization, while biparatopic sdAbs may induce receptor dimerization and/or oligomerization, leading to faster receptor internalization [22,29]. 4A10-3G7 could induce PDGFβ receptor oligomerization leading to faster internalization speed than 4A10 alone. It may be that 3G7-3G7 cannot induce receptor dimerization due to the linker length of the bivalent construct or the binding epitope of 3G7. This could explain the similar internalization speed as the monomeric construct 3G7 and substantiates our suggestion that the binding epitope of the targeting moiety can influence the internalization rate. 

Importantly, we show that the internalization rate reflects the intracellular accumulation after a longer period of time: targeting moieties with higher internalization rates transport more cargo into the cell. That excludes the efficient recycling back to the membrane, which has for instance been described for HER2-targeted ADC Kadcyla [30]. This is in line with other studies, including one study in which surface depletion of the fast internalizing target antigen is observed [28]. This may also be an explanation for the stabilization of the intracellular accumulation we observed.

When the internalization kinetics of the different sdAb constructs was investigated, it was determined that their binding does not interfere with the activation status of the PDGFβ receptor. We identified a possible route of sdAb internalization, showing that all sdAbs internalize mainly via macropinocytosis and end up in the lysosomes, the ideal destination for intracellular drug delivery [3,4,31]. Macropinocytosis of the PDGFRβ complex has been described before, but here macropinocytosis is induced without phosphorylating the receptor [32,33].

Conjugated to the cytotoxic payload Auristatin F (AF), the sdAb-Lx-AF-conjugates with faster internalization rates displayed more cytotoxicity than slower internalizing AF-conjugates. In other studies fast internalizing sdAb constructs were also more potent in vitro, although not much attention has been given to these findings before [12,13]. When the same assay was performed in presence of vacuolin-1, a lysosomal exocytosis inhibitor [24], none of the conjugates showed any cell killing. This shows that the sdAbs deliver drugs via the endosomal-lysosomal degradation pathway, making them interesting candidates for intracellular drug delivery [34]. 

## 5. Conclusions

As a proof-of-principle, we have designed four different sdAb constructs with similar binding affinities to PDGFRβ but with different internalization rates resulting in different cytotoxicity level when used as sdAb drug-conjugates. sdAbs are versatile targeting moieties which can be explored for effective drug conjugate development. 

## Supplementary Materials

The following are available online at https://www.mdpi.com/article/10.3390/biom11070927/s1, Figure S1: PDGFβ receptor quantification on SCCVII-rPDGFRβ cells, Figure S2: Bacterial periplasm containing sdAbs binding to ectodomain, Figure S3: Monomeric sdAbs binding to ectodomain, Figure S4: Competition assay with 3G7-IRDye800, Figure S5: Purity of IRDye800 conjugated sdAbs on a SDS-PAGE gel, Figure S6: Serial dilutions of sdAbs binding to immobilized rat PDGFRβ ectodomain, Figure S7: Original blots and quantification of Figure 4, Figure S8: Deconvoluted mass spectra obtained upon SEC-MS analysis of the different auristatin F (AF) conjugates, Figure S9: Cell viability assay on SCCVII cells, Video S1. Live imaging of internalizing 4A10-3G7, Table S1: Calculated immune library sizes.
