# Supplementary material for: Single Domain Antibodies as Carriers for Intracellular Drug Delivery: A Proof of Principle Study
Source: Biomolecules. 2021 Jun 22;11(7):927. doi: 10.3390/biom11070927 (PMC8301836; doi:10.3390/biom11070927)
Supplement: Supplementary file 1 [file biomolecules-11-00927-s001.zip › supp revised/Pronk_Biomolecules Supplementary 2021-06-07 revised.pdf]

Single domain antibodies as carriers for intracellular drug delivery: a proof of principle study

Sebas D. Pronk [1], Erik Schooten [1], Jurgen Heinen [2], Esra Helfrich [2], Sabrina Oliveira [2,3,*] and Paul M. P. van Bergen en Henegouwen [2]

[1] Research and Development Department, LinXis BV, Amsterdam, The Netherlands; pronk@linxispharmaceuticals.com, schooten@linxispharmaceuticals.com
[2] Cell Biology, Neurobiology and Biophysics, Department of Biology, Science Faculty, Utrecht University, Utrecht, The Netherlands; jurgen.heinen@student.uva.nl; esra.helfrich@student.hu.nl; s.oliveira@uu.nl; p.vanbergen@uu.nl
[3] Pharmaceutics, Department of Pharmaceutical Sciences, Science Faculty, Utrecht University, The Netherlands
* Correspondence: s.oliveira@uu.nl; Tel.: +31 6 34103460

Supplementary Figures and Tables Content

- Figure S1. PDGFβ receptor quantification on SCCVII-rPDGFRβ cells.
- Table S1. Calculated immune library sizes.
- Figure S2. Bacterial periplasm containing sdAbs binding to ectodomain.
- Figure S3. Monomeric sdAbs binding to ectodomain.
- Figure S4. Competition assay with 3G7-IRDye800.
- Figure S5. Purity of IRDye800 conjugated sdAbs on a SDS-PAGE gel.
- Figure S6. Serial dilutions of sdAbs binding to immobilized rat PDGFRβ ectodomain.
- Figure S7. Original blots and quantification of Figure 3.
- Figure S8. Deconvoluted mass spectra obtained upon SEC-MS analysis of the different auristatin F (AF) conjugates.
- Figure S9. Cell viability assay on SCCVII cells.

Supplementary Figures and Tables

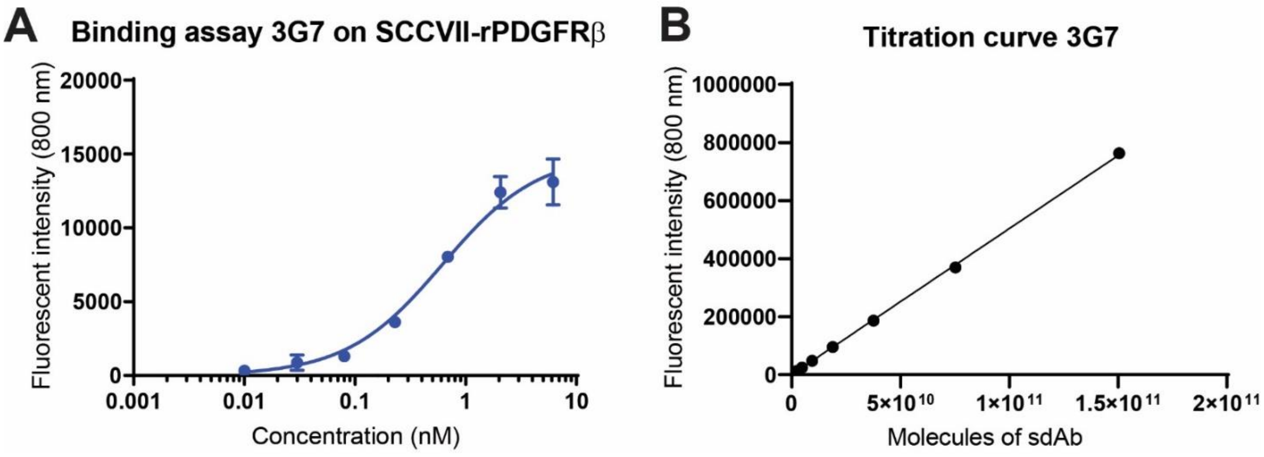

Figure S1. PDGFβ receptor quantification on SCCVII-PDGFRβ cells. (**A**). A binding assay with 3G7 conjugated to a fluorophore was performed in order to determine the B_{max}. (**B**). The B_{max} was then interpolated in the titration curve of 3G7 to determine the number of sdAb molecules at B_{max}.

Table S1. Calculated immune library sizes.

Animal	Library	Size
SNL154	rP-154	$9.6 * 10^6$
SNL155	rP-155	$4.8 * 10^6$

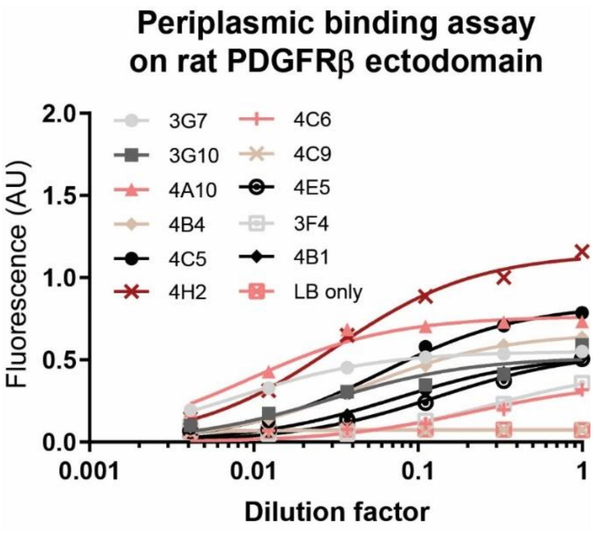

Figure S2. Serial dilutions of unpurified bacterial periplasm containing sdAbs binding to immobilized rat PDGFRβ ectodomain.

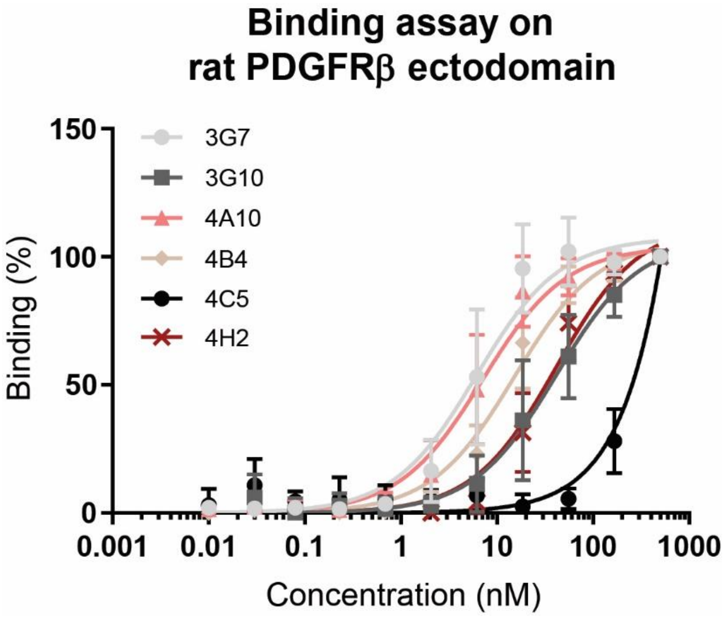

Figure S3. Serial dilutions of monomeric sdAbs binding to immobilized rat PDGFRβ ectodomain.

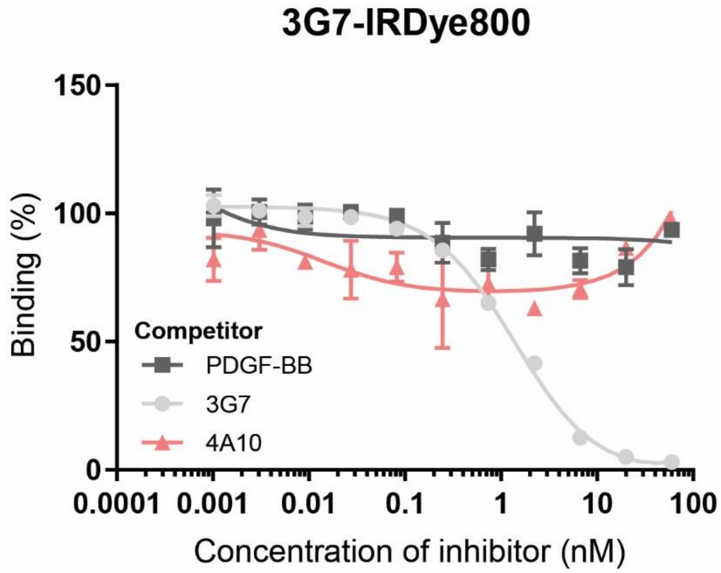

Figure S4. Competition assay in which 3G7 conjugated to IRDye800 (3G7-IRDye800) was incubated on immobilized rat PDGFRβ-ECD in the presence or absence of excess unconjugated competitor (3G7 or PDGF-BB).

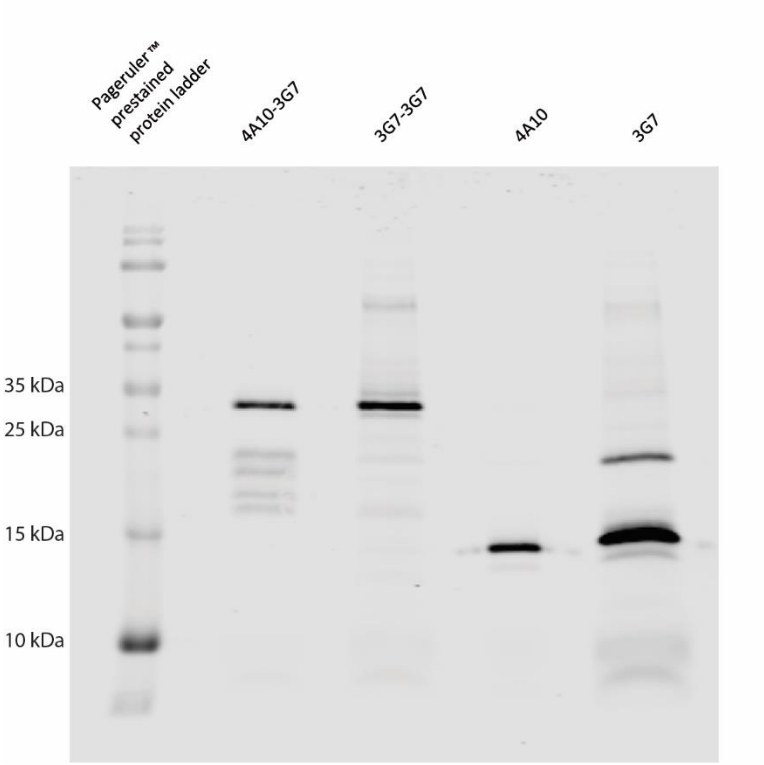

Figure S5. Purity of IRDye800 conjugated sdAbs on a SDS-PAGE gel. Signal shown is the IRDye800 signal.

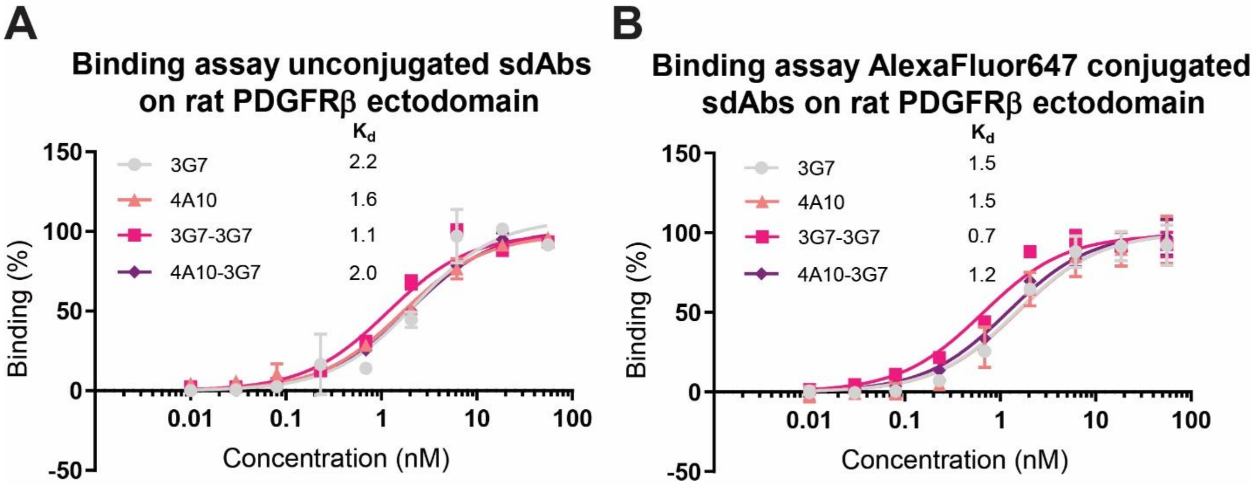

Figure S6. Serial dilutions of sdAbs binding to immobilized rat PDGFRβ ectodomain. (**A**). Binding assay of unconjugated sdAbs binding to immobilized rat PDGFRβ ectodomain detected indirectly. (**B**). Binding assay of AlexaFluor647 conjugated sdAbs binding to immobilized rat PDGFRβ ectodomain detected indirectly.

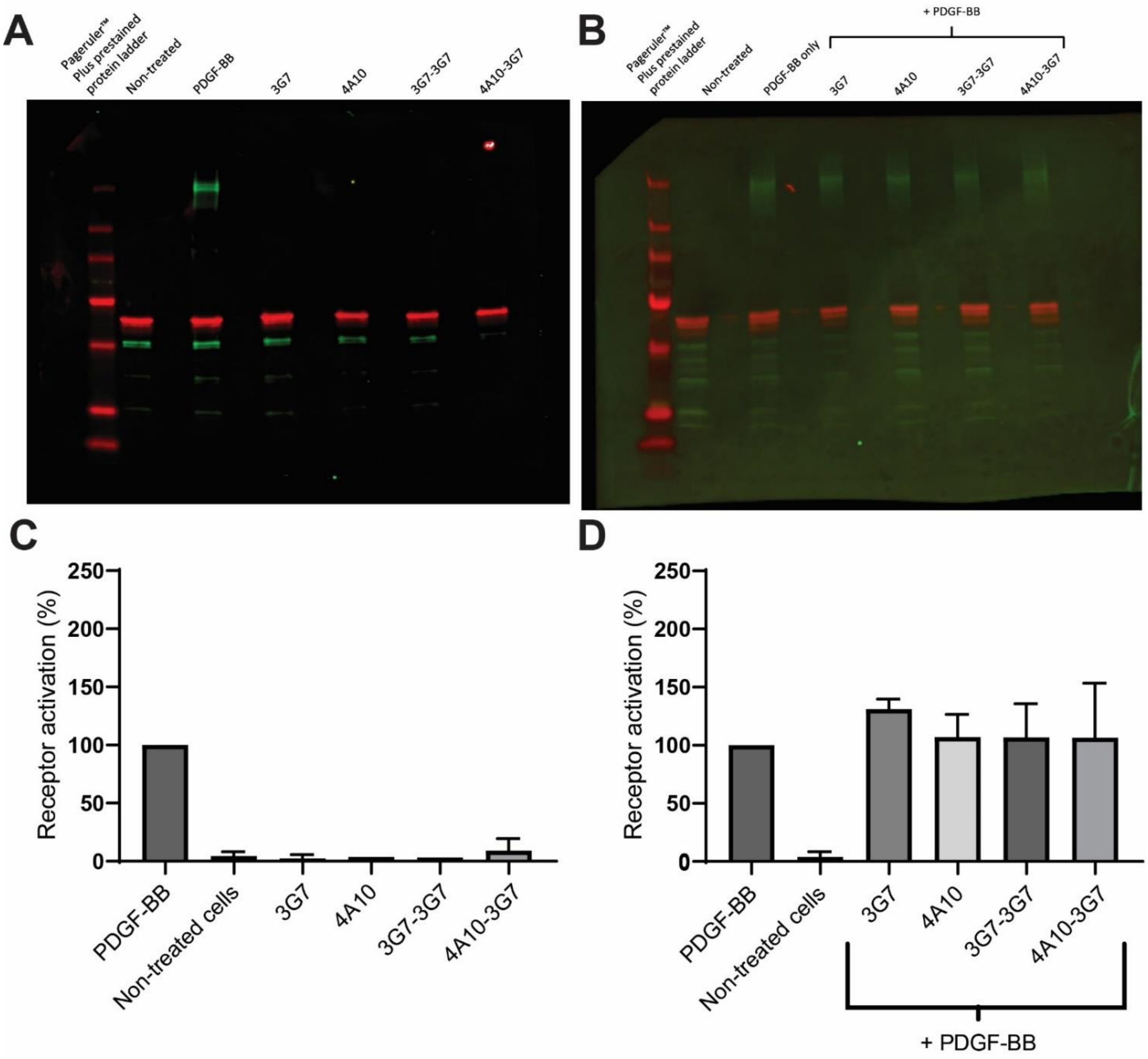

Figure S7. Original blots and quantification of Figure 4. Displayed in red is the IRDye700 signal (actin), and in green the IRDye800 signal (phosho-PDGFRβ). (**A**). Untouched and uncropped blot of Figure 4A. (**B**). Untouched and uncropped blot of Figure 4B. (**C**). Quantification (phospho-PDGFRβ / actin) of performed activation assays. (**D**). Quantification (phospho-PDGFRβ / actin) of performed activation inhibition assays.

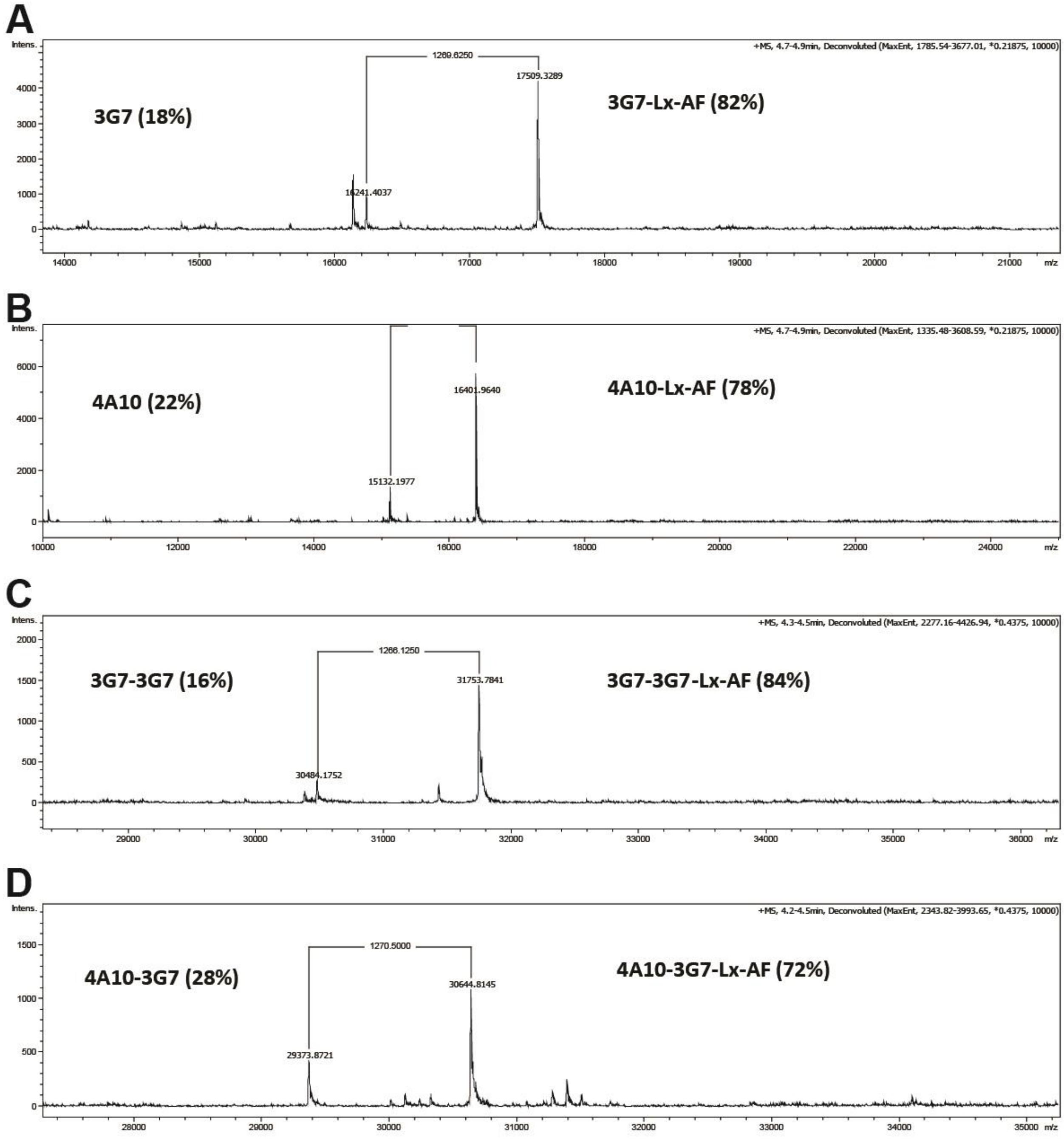

Figure S8. Deconvoluted mass spectra obtained upon SEC-MS analysis of the different auristatin F (AF) conjugates. (**A**). SEC-MS of 3G7 after conjugation to AF. (**B**). SEC-MS of 4A10 after conjugation to AF. (**C**). SEC-MS of 3G7-3G7 after conjugation to AF. (**D**). SEC-MS of 4A10-3G7 after conjugation to AF.

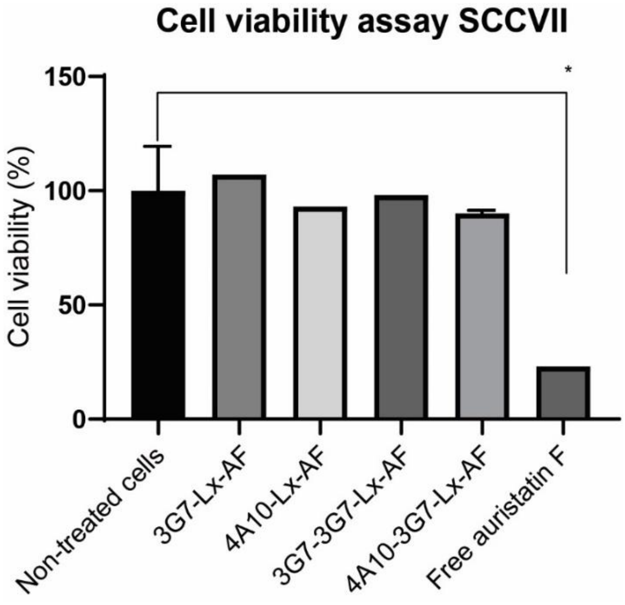

Figure S9. Cell viability assay on SCCVII cells. Cell viability is measured after 72 hours of treatment with 250nM conjugates or free Auristatin F. P values were determined using a one-way ANOVA test. * = P≤0.05 and ns = not significant.
